# Risk of Pharmacological or Hospital Treatment for Depression in Patients with Colorectal Cancer–Associations with Pre-Cancer Lifestyle, Comorbidity and Clinical Factors

**DOI:** 10.3390/cancers13081979

**Published:** 2021-04-20

**Authors:** Trille Kristina Kjaer, Ida Rask Moustsen-Helms, Vanna Albieri, Signe Benzon Larsen, Thea Helene Degett, Anne Tjønneland, Christoffer Johansen, Susanne K. Kjaer, Ismail Gogenur, Susanne Oksbjerg Dalton

**Affiliations:** 1 Survivorship and Inequality in Cancer Department, Danish Cancer Society Research Center, 2100 Copenhagen, Denmark; irmh@ssi.dk (I.R.M.-H.); theahd@cancer.dk (T.H.D.); christoffer.johansen@regionh.dk (C.J.); sanne@cancer.dk (S.O.D.); 2 Department of Infectious Disease Epidemiology and Prevention, Statens Serum Institut, 2300 Copenhagen, Denmark; 3 Statistics and Data Analysis Department, Danish Cancer Society Research Center, 2100 Copenhagen, Denmark; vanna@cancer.dk; 4 Department of Urology, Copenhagen Prostate Cancer Center, Copenhagen University Hospital-Rigshospitalet, 2100 Copenhagen, Denmark; signe.liv.benzon.larsen@regionh.dk; 5 Center for Surgical Science, Zealand University Hospital, 4600 Køge, Denmark; igo@regionsjaelland.dk; 6 Diet, Genes and Environment Department, Danish Cancer Society Research Center, 2100 Copenhagen, Denmark; annet@cancer.dk; 7 Department of Public Health, University of Copenhagen, 1014 Copenhagen, Denmark; 8 Late Effect Research Unit CASTLE, Finsen Center, Copenhagen University Hospital-Rigshospitalet, 2100 Copenhagen, Denmark; 9 Virus, Lifestyle and Genes Department, Danish Cancer Society Research Center, 2100 Copenhagen, Denmark; susanne@cancer.dk; 10 Department of Gynecology, Copenhagen University Hospital-Rigshospitalet, 2100 Copenhagen, Denmark; 11 Danish Colorectal Cancer Group, 2100 Copenhagen, Denmark; 12 COMPAS, Danish Research Center for Equality in Cancer, Oncology Department and Palliative Units, Zealand University Hospital, 4700 Naestved, Denmark

**Keywords:** colorectal cancer, depression, late effects, cancer survivorship

## Abstract

**Simple Summary:**

Depression is a common disorder in cancer patients. In this population-based prospective cohort study, we investigated if patients with colorectal cancer experience a higher risk of pharmacological or hospital treatment for depression than age- and gender-matched cancer-free comparison persons when differences in lifestyle, anthropometry, socioeconomic position or comorbidity where taken into account. To identify potential risk factors, we further investigated the associations between depression and antidepressant use and pre-cancer lifestyle factors, clinical factors and treatment factors. The study results may help point toward vulnerable groups of patients at risk of depression.

**Abstract:**

We investigated the risk of depression in colorectal cancer (CRC) patients and associated risk factors. The 1324 patients with CRC and 6620 matched cancer-free participants from the Diet, Cancer and Health study were followed for up to 16 years for either a first hospitalization for depression or antidepressant prescription after diagnosis of CRC cancer or study entry date. Information on the outcome and covariates was retrieved from the Danish Colorectal Cancer Group database, the national health registries and questionnaires. Cumulative incidence of depression was estimated, and Cox regression models were used to evaluate the association between risk factors and depression incidence. During follow-up, 191 (14.4%) patients with CRC and 175 (2.6%) cancer-free comparison persons experienced depression. After adjustments, in the first year after cancer diagnosis, patients with CRC had a 12-fold higher hazard compared with the cancer-free population (HR, 12.01; 95% CI, 7.89–18.28). The risk decreased during follow-up but remained significantly elevated with an HR of 2.65 (95% CI, 1.61–4.36) after five years. Identified risk factors were presence of comorbidities, advanced disease stage and use of radiotherapy, while life style factors (pre-cancer or at diagnosis) and chemotherapy did not seem to contribute to the increased risk.

## 1. Introduction

Due to the enhanced screening and prevention efforts and improved treatments in high-income countries, the mortality rates for colorectal cancer (CRC) have decreased [1]. Survivors after colorectal cancer often suffer from late effects from the cancer and its treatments [2,3] that may affect their wellbeing and quality of life [4,5].

The physical late effects of CRC and its treatments are well known and include among other things gastrointestinal and urogenital problems [6], sexual dysfunction [7] and neuropathy [8,9]. This group of patients may also live with a colostomy as a result of the surgical intervention, giving rise to specific problems. Additionally, emotional and psychological late effects such as fear of recurrence, negative body image, anxiety and depression have been reported in a few previous reports [2,3], but research is limited [10]. Evidence suggests that patients with CRC do experience increased levels of self-reported depressive symptoms and depression compared with comparisons without cancer [11,12]. In a prospective Dutch study among 2625 CRC patients with stage I–IV registered in the PROFILES registry, significantly more of the patients (19%) reported having a depression compared with 13% among an age- and sex-matched normative population (*n* = 315) [12]. Other studies report a prevalence ranging from almost none to 47% of patients reporting depressive symptoms [11].

Less is known about whether patients with CRC actually are diagnosed (using objective measures) with a clinically relevant depression that requires medical attention, and the body of evidence is even sparser for identifying patient-specific risk factors for depression in this group of cancer patients.

In this prospective cohort study with up to 16 years of follow-up, we used objective measures of depression, defined as either psychiatric admission or outpatient contact for depression or as prescription of anti-depressants, to investigate if patients with CRC experience a long-term increased risk of depression compared with the background population. In a within-cohort analysis among patients with CRC, we further investigated the associations between depression and socio-demographics, lifestyle factors and cancer-specific as well as treatment factors.

## 2. Materials and Methods

### 2.1. Study Population

We used data from the Diet, Cancer and Health (DCH) study, a large prospective cohort study conducted between December 1993 and May 1997 among 56,506 persons who lived in the greater Copenhagen and Aarhus areas in Denmark. The study has been described in detail previously [13]. In short, to be invited to the study participants had to be 50–65 years old, be born in Denmark and have no previous history of cancer, excluding non-melanoma skin cancer. At enrollment, the participants filled in a comprehensive questionnaire covering diet, lifestyle and physical exercise habits. Anthropometrical measurements such as height and weight were conducted by a lab technician, and biological material was collected [13].

For the present study, we used the unique Danish personal identification numbers to link the DCH cohort to the Central Population Registry [14] to obtain information on death and emigration. Next, we linked the cohort to the Danish Colorectal Cancer Group (DCCG.dk) database [15] to identify participants who were diagnosed with CRC (ICD 10 code C18–20) in the period from 1 January 2001 to 31 December 2016. The DCCG.dk is a clinical quality database and contains information on all patients diagnosed with CRC in Denmark since 2001 and includes information on clinical factors and treatment information including date and type of surgery. Lastly, we linked the cohort to the Danish Cancer Registry, which holds information on all individuals with cancer since 1943 [16].

Cancer-free persons participating in the DCH study were used as comparison persons in a 1:5 matched design. The matching was done at the date of the CRC diagnosis or surgery (study entry date), and the matching variables were age, sex and time since entry into the DHC study.

**Figure 1:**
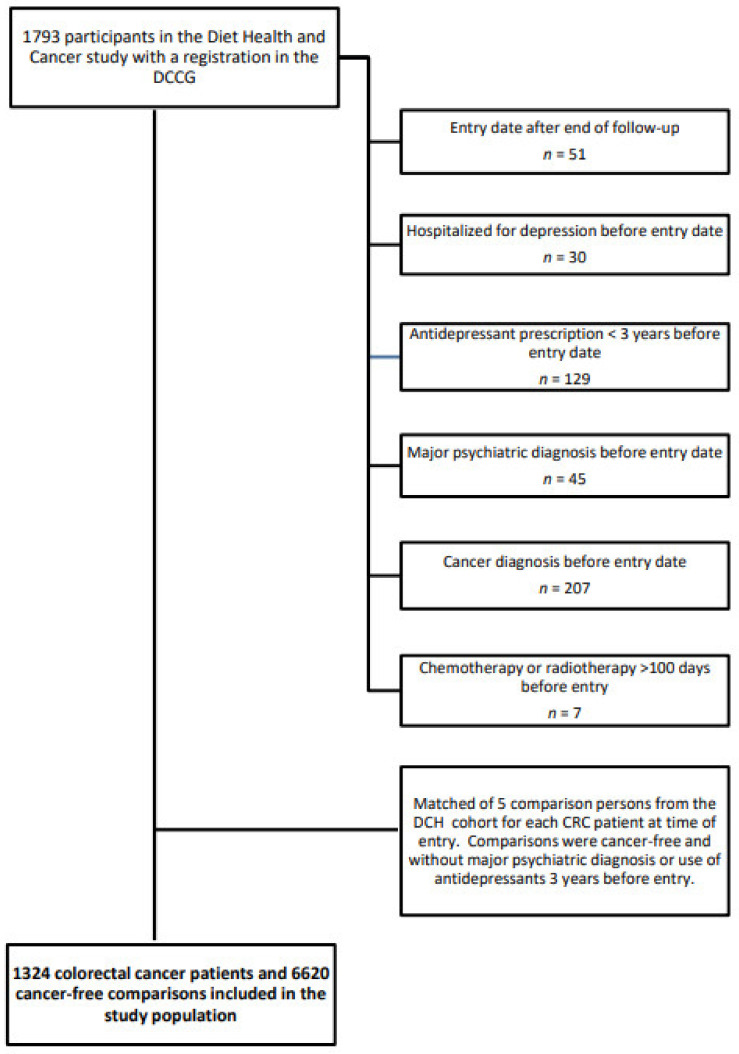
Flowchart of the formation of the study population of 1324 colorectal cancer patients and 6620 cancer-free comparison persons who participated in the Diet, Cancer and Health study, 2001–2016.

People who had been hospitalized for depression or other major psychiatric events, had redeemed a prescription for an antidepressant up to three years before inclusion or were diagnosed with any cancer prior to the CRC diagnosis or study entry were excluded. After exclusions, data on 1324 patients with CRC and 6620 cancer-free comparison persons were included in the analyses (Figure 1).

All participants in the study were followed from either the date of diagnosis of colorectal cancer or study entry for the matched comparison persons until date of hospitalization for depression or date of first redeemed prescription of an antidepressant, date of hospitalization for other major psychiatric event, diagnosis of a new primary cancer or a cancer diagnosis in the comparison person, emigration, death, or end of follow-up (31 December 2016), whichever came first.

The Diet, Cancer and Health study was approved by the regional ethical committees in Copenhagen and Aarhus ((KF) 11-037/01), and the present study was approved by the Danish Data Protection Agency (2013-41-4232).

### 2.2. Information on Hospitalization for Depression and Redeemed Prescription for an Antidepressant

To obtain information on incident in- or outpatient hospital contact for unipolar depression (ICD-10: F32-33) we linked information from each person with CRC and cancer-free comparison person to the files of the National Patient Register (NPR). Since its establishment in 1977, the Register contains individual information on all diseases leading to hospitalization (ICD-8 and ICD-10) and their treatments and, since 1995, outpatient visits and in- and outpatient contacts to psychiatric hospitals in Denmark [17].

From the Danish National Prescription Registry (DNPR), we obtained date of redeemed prescriptions of antidepressant within group N06A of the Anatomic Therapeutic Chemical (ATC) classification system. DNPR holds individual information on all drugs redeemed with a prescription at Danish pharmacies [18].

### 2.3. Covariates

Clinical and treatment information was retrieved from the DCCG.dk database and included information on type of cancer (colon or rectal), disease stage (I–IV), ASA score (I–IV or unknown), date of surgery and procedures (elective or acute surgery), stoma (yes/no) and surgery complications (yes/no). ATC codes for radiation and chemotherapy and dates for each radiotherapy (RT) treatment and cycle of chemotherapy (CT) were obtained from the NPR. RT administered within 100 days before or after date of surgery was considered neo-adjuvant or palliative therapy, respectively. Neo-adjuvant CT was defined as receiving CT 100 days before surgery, and adjuvant CT was defined as beginning CT within 180 days after surgery with less than 3 months between cycles.

From the NPR we also retrieved information on dates and diagnosis of comorbid conditions up to 3 years prior to study entry. We used modified Charlson Comorbidity Index (CCI) scores [19] to measure comorbidity by adding up to 19 somatic conditions, excluding cancer. The CCI quantifies the impact of somatic disease on survival. Based on the total number of disorders, each disorder is assigned a weighted score from 1–6 according to severity, and an accumulated score is calculated for each person [19].

Information on highest attained level of education (‘short’ 7–9 years, mandatory school; ‘medium’ 8–12 years, upper secondary or vocational education; or ‘long’ >12 years, higher education) and cohabitation status (married/cohabitating or living alone) was obtained from the Danish administrative and social registries administered by Statistic Denmark [14,20].

Questionnaire information and anthropometry measurements from the DCH cohort at time of enrollment were used to identify the pre-cancer smoking (current, former, never); alcohol consumption, categorized as 0 (abstainers), 1–36 (moderate use) and >36 g/day for men and 0, 1–24 and >24 g/day for women (excessive use); metabolic equivalents (MET)-scores in quartiles; and body mass index (BMI), categorized as underweight (<18.5 kg/m^2^), normal weight (18.5–24.9 kg/m^2^), overweight (25–29.9 kg/m^2^) or obese (≥30 kg/m^2^).

### 2.4. Statistical Analyses

The primary outcome of interest was depression, defined as either a first hospitalization for depression or an antidepressant prescription.

Cumulative incidence function analyses were used to estimate the incidence of depression, taking death into account as a competing event.

Cox proportional hazard regression models were applied to estimate the hazard ratio (HR) and 95% confidence intervals (CIs) for pharmacological or hospital treatment for depression among patients with CRC compared with the matched comparison persons with time since diagnosis as the underlying timescale. Three models were performed. In the first model, we adjusted for age, sex and time since entrance in DHC (modeled as restricted cubic spline). In the second model, we included education, pre-cancer lifestyle factors (smoking, alcohol, BMI and MET-score) to adjust for potential confounding by these characteristics and in the final model, we further included as possible mediators comorbidity (CCI) (time varying), cohabitation (time varying) and disease stage.

Within-cohort analyses among patients with CRC were used to investigate risk factors associated with pharmacological or hospital treatment for depression in unadjusted and multivariate Cox proportional hazard regression models with time since diagnosis as the underlying timescale. The following risk factors were included: pre-cancer lifestyle factors (smoking, alcohol consumption, BMI and MET-score), comorbidity and cohabitation status (both time varying), clinical factors (cancer type and disease stage at time of diagnoses) and treatment factors (surgery, radiotherapy and chemotherapy, all time varying).

The proportional hazard assumption was evaluated by testing the correlation coefficient for transformed time and scaled Schoenfeld residuals and associated plots. In the patients with CRC and cancer-free comparison cohort, the assumption of proportionality was violated so the overall effect was evaluated as time-dependent and estimated in four intervals: 0–1 year, 1–3 years, 3–5 years and more than 5 years.

Two sets of sensitivity analyses were performed. The first sensitivity analysis investigated whether advanced disease stage could be considered as the main explanation for the risk of depression. We thus included only CRC patients with stage I to III and their matched cancer-free comparison persons and re-computed the main analysis and the within cohort analyses, adjusting for the same confounders and mediators as in the first models. The analyses were repeated separately in colon and rectum cancer patients. The second sensitivity analysis was performed only on the within-cohort among patients with CRC, using information on smoking, alcohol intake and BMI at time of CRC diagnosis as reported to the DCCG.dk database.

All statistical analysis was done in R version 3.5.1 packages ’survival’, ’etm’, ’ggplot2 [21].

## 3. Results

The 1324 patients with CRC and 6620 matched comparison people contributed with 5549 and 38,753 person years, with a median follow-up of 2.7 years and 4.9 years (range 0–16 years), respectively. The distribution of sociodemographic and lifestyle characteristics was similar among patients with CRC and cancer-free comparison persons except that more patients with CRC were current smokers and had comorbidity at entry (Table 1). The majority of the 879 colon cancer patients and the 445 rectum cancer patients received surgery (90% and 87%, respectively) (Table 2).

**Figure 2:**
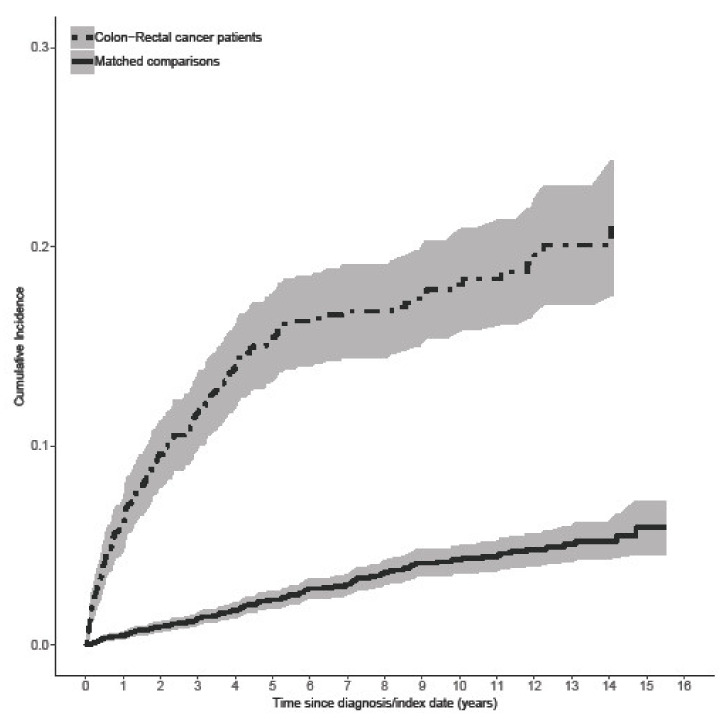
Cumulative incidence functions of hospitalization for depression or antidepressant prescription in 1324 patients with colorectal cancer and 6620 matched cancer-free comparison persons.

A total of 191 (14%) patients with CRC and 175 (3%) cancer-free comparison persons experienced depression. The cumulative incidence function showed an increased probability of experiencing depression among patients with CRC throughout the 16-year study period, with the steepest increase in the first five years after entry. At five years, the cumulative incidence for depression was 15.4% for patients with CRC compared with 2.3% for comparisons (Figure 2).

After adjustments, patients with CRC had significantly higher risk of depression than cancer-free comparison persons in all studied periods. Investigating the pattern of depression during the follow-up period, we found significantly higher HRs in the first year after entry compared with the cancer-free comparison persons (HR, 12.01; 95% CI, 7.89–18.28), followed by a slight decrease in the following intervals (1–3 years: HR, 8.40; 95% CI, 5.65–12.49, 3–5 years: HR, 5.77; 95% CI, 3.60–9.25, and after more than five years: HR, 2.65; 95% CI, 1.61–4.36) (Table 3). Sensitivity analyses, including only patients with CRC with disease stage I–III, reduced the estimates with an adjusted HR of 7.93 (95% CI, 4.77–13.19) after the first year, an HR of 6.90 (95% CI, 4.30–11.09) after 1–3 years, an HR of 6.04 (95% CI, 3.60–10.13) after 4–5 years and an HR of 2.34 (95% CI, 1.38–3.98) after more than five years. Separate analyses on each cancer site showed that colon cancer patients had higher hazards than rectum cancer patients (data not shown).

Within-cohort analyses showed that only CCI scores of 1-2 (HR, 1.74; 95% CI, 1.24–2.43) and CCI scores of 3 or more (HR, 2.74; 95% CI, 1.84–4.09), stage 4 disease (HR, 3.07; 95% CI, 1.95–4.83) and radiotherapy treatment (HR, 2.76; 95% CI, 1.82–4.19) were associated with depression in the fully adjusted models (Table 4). Sensitivity analyses including only patients with CRC with stages I–III showed no notable effects on the HRs observed (Table S1), neither did using lifestyle indicators at time of entry in the DCCG.dk database (Table S2).

## 4. Discussion

In this prospective cohort study among participants in the DCH study, we found a more than 12-fold higher risk of depression among patients with CRC compared with the matched cancer-free population in the first year after a CRC diagnosis or study entry. The risk of depression decreased during follow-up but remained statistically significant and more than 2-fold higher than in the matched cancer-free population beyond 5 years since diagnosis. Risk factors for depression included presence of comorbidities, advanced disease stage and use of radiotherapy, while chemotherapy or lifestyle factors did not seem to contribute to the increased risk.

To our knowledge, this is one of only a few studies investigating objectively defined depression, where assessment by a physician warrants either pharmacologically or specialized psychiatric in- or out-patient treatment in patients with CRC compared with matched cancer-free comparison persons. Sun and colleagues [22] investigated the risk of mood disorders including depression among patients with CRC (*n* = 27,242) and matched cancer-free persons (*n* = 108,046) in a large Taiwanese population-based study using medical claim records (ICD-9-CM codes) registered in the Taiwan National Health Insurance Research Database covering 99% of the Taiwan residents. Patients with CRC had an overall increased risk of depressive disorders (HR, 1.54; 95% CI, 1.39–1.71) after adjustments for socioeconomic factors and comorbidity and stratified analyses revealed that the risk was most pronounced among the patients with more than one year of follow-up (HR, 1.14; 95% CI, 0.99–1.32) than within the first year of follow-up (HR, 0.77; 95% CI, 0.66–0.91). The authors did not have information on stage but found no association between radiotherapy, chemotherapy or colostomy and depressive disorders [22]. Although the results of this study correspond with the present study, the two studies may not be directly comparable as cultural differences and perception of depressive disorders in Taiwan may differ from that of Western countries, with resulting relatively lower lifetime rate for major depression in the general Taiwanese population [22].

In another large study, general practice consultations and prescribing behavior in relation to depression and anxiety were compared between long-term (>5 years) cancer patients after three common cancers, including 5068 patients with CRC and 20,128 matched cancer-free control patients registered in the General Practice Research Database holding diagnostics, test results and prescription data from 450 general practices in the UK [23]. Only 8% of patients with CRC consulted for depression during follow-up, while 17% of the patients were prescribed an antidepressant. Contrary to our findings, the multivariate models in this study, including comorbidity or previous history of depression, showed no significant differences in general practice consultations for depression between patients with CRC and matched controls (OR, 1.07; 95% CI, 0.94–1.22) or in being prescribed antidepressants (OR, 1.06; 95% CI, 0.96–1.17) during the three years of follow-up. However, it was not possible to distinguish between patients with clinically significant depression and those who merely consulted for depression, whereby mild or non-cases may have been included [23]. In our study, we were able to include both severe cases of depression requiring hospitalizations or outpatient psychiatric treatment as well as moderate/severe cases requiring pharmacological treatment. A hospital diagnosis for depression is considered valid and has a 99% specificity in the general Danish population [24]. Likewise, antidepressants are available to Danish patients by prescription only and may therefore be a valid proxy for pharmacologically treated moderate to severe depression.

In a previous study, we observed the risk of hospitalization for affective disorders in a large Danish sample of cancer patients, including patients with CRC [25]. At that time, we reported increased relative risk estimates (RR) across all cancer types, sex and disease extent, when compared with the entire cancer-free Danish background population. The patients were followed for up to 30 years (1973–2003), but the overall excess risk was highest in the first year after the diagnosis. For male patients with CRC, the RR was 2.14 (95% CI, 1.67–2.75) in the first year after diagnosis, and the risk remained increased in the following years although statistically insignificant. For female patients with CRC, the risk was only elevated in the first few follow-up years with RRs of 1.16 (95% CI, 0.90–1.51) and 1.28 (95% CI, 1.08–1.52) after 1 and 1–4 years, respectively. Contrary to the present study, only hospitalizations, and thus very severe cases of depression, were investigated which may explain the relatively lower risk than found in this study.

Lower rates of depression, relative to our study, were found in a large cohort (*n* = 56,182) of elderly (≥65 years) patients with CRC using SEER-Medicare data and ICD-9 codes for diagnosis of mental disorders. Diagnostic rates for all depressive disorders ranged from 1.5% to 1.8% over five years and the rates were significantly lower for patients with CRC than the non-cancer comparison persons throughout the investigated period. However, the authors suggested a possible under-recognition of depression in their study population and found more than 70% of the patients were diagnosed with depressive disorder NOS (Not Otherwise Specified), indicating that current diagnostic criteria may induce difficulties in recognizing depression in patients with CRC [26].

Strengths of our study include the use of participants in a prospective cohort study, population-based registries with medically validated information on hospital diagnoses and prescriptions, the longitudinal design with up to 16 years of follow-up and with no loss to follow-up due to precise linkage using the unique personal identification number assigned to all Danish citizens at birth as key identifier. We were able to include comprehensive clinical data at time of diagnosis and data on pre-cancer lifestyle factors at time of inclusion in the DHC cohort. We included cohabitation and comorbidity as time-varying variables, thereby enhancing the robustness of our results.

A limitation of our study is the specificity of the outcome measured as pharmacological or hospital treatment for depression combined. Hospital admission for depression is rare and only occurs in severe cases and may therefore not capture the full range of depressive episodes in the cohort. By applying receipt of antidepressant medication, mostly prescribed in general practice, as a proxy for pharmacologically treated depression, we included moderate to severe depression treated by general practitioners or practicing psychiatrists in our outcome measure. Persons with mild depression or moderate/severe depression who were undiagnosed or opted for psychotherapeutic treatment were not included, however, and it is likely that surveillance of patients with CRC may result in differences in awareness and treatment of depression between cancer patients and comparisons. Further, misclassification of antidepressants as a measure of depression may be present as we did not have information on indications for antidepressant treatments that are also used for treatment of, e.g., anxiety or pain. Previous studies show that 10–15% of the prescribed antidepressant are given for anxiety and 10–30% for non-psychiatric conditions such as pain [27,28,29], in which especially the latter indication may be a source of bias.

Another limitation of our study is the selective population in the DHC study, where participants were more likely to have had longer lengths of education and better overall health than invited people who chose not to participate in the study [13,30]. This may affect the generalizability of our results. Finally, we did not have information on disease recurrence or progression, which may likely be associated with depression, and occurrence of these events may contribute to the persistently increased risk of depression for years after diagnosis and primary treatment.

## 5. Conclusions

Patients with CRC have a long-term significantly higher risk of depression than matched cancer-free comparison persons. Clinicians should be aware of this late effect of CRC and its treatments, particularly in patients with comorbid conditions, advanced disease stage or patients who are treated with radiotherapy.

## Figures and Tables

**Table 1:** Sociodemographic and comorbidity at time of study entry and lifestyle factors reported at time of enrollment among 1324 patients with colorectal cancer and 6620 matched comparison persons at time of enrollment in the Danish Diet, Cancer and Health study.

	Patients with Colorectal Cancer *n* (%)	Cancer-Free Comparison Persons *n* (%)
Mean age at study entry (SD)	71.3 (5.98)	71.3 (5.97)
Sex		
Women	563 (43)	2815 (43)
Men	761 (58)	3805 (58)
Education ^1^		
Long	337 (26)	1845 (28)
Medium	743 (57)	3551 (54)
Short	231 (18)	1145 (18)
Cohabiting ^2^		
Yes	903 (68)	4479 (68)
No	421 (32)	2141 (32)
Charlson Comorbidity Index Score ^3^		
0–1	843 (64)	4613 (70)
2	377 (29)	1691 (26)
≥3	104 (8)	316 (5)
Smoking		
Current	481 (36)	2025 (31)
Former	431 (33)	2171 (33)
Never	411 (31)	2415 (37)
Body Mass Index		
<25 kg/m^3^	506 (38)	2866 (43)
25–30 kg/m^3^	600 (45)	2854 (43)
>30 kg/m^3^	217 (16)	896 (14)
Alcohol consumption per week ^4^		
Abstainers	83 (6)	426 (7)
Moderate use	1016 (77)	5188 (79)
Excessive use	218 (17)	977 (15)
Physical activity (MET-score) ^5^		
4th quartile (most active)	322 (24)	1657 (25)
3rd quartile	327 (25)	1739 (26)
2nd quartile	347 (26)	1603 (24)
1st quartile (least active)	326 (25)	1604 (24)

Percentages are rounded to whole numbers. ^1^ Short education: 7–9 years, mandatory school; medium education: 8 to 12 years, upper secondary high school or vocational education; higher education: >12 years, higher education. ^2^ Married/cohabitating or living alone. ^3^ Comorbidity is classified according to a modified the Charlson Comorbidity Index (CCI), excluding cancer. ^4^ Based on recommendations from the Danish Health Authorities at time of inclusion. Moderate use, 1–14 drinks per week for women and 1–21 drinks per week for men. Excessive use, >14 drinks per week for women and >21 drinks per week for men. ^5^ The metabolic equivalent of task, i.e., a measure of energy cost of physical activities. Calculated as kcal/kg/hour from an average of summer and winter physical activity multiplied by number of hours per week. 1st quartile: <=14.5 (least active), 2nd quartile: 14.5–26, 3rd quartile: >26–43.8, 4th quartile (most active): >43.8.

**Table 2:** Disease and treatment characteristics in 1324 patients with colorectal cancer, by cancer type.

	Cancer Type	
	Colon	Rectum
	*n* = 879 (%)	*n* = 445 (%)
Disease stage		
I	119 (14)	97 (24)
II	302 (36)	99 (24)
III	228 (27)	121 (30)
IV	186 (22)	90 (22)
Surgery ^1^		
No	90 (10)	59 (13)
Yes	789 (90)	386 (87)
Surgical complications ^1^		
Yes	404 (46)	139 (30)
Stoma ^1^		
Yes	87 (10)	203 (46)
Chemotherapy ^1^		
Yes	370 (42)	192 (43)
No	509 (60)	253 (57)
Radiotherapy ^1^		
Yes	68 (8)	149 (34)
No	811 (92)	296 (67)

Percentages are rounded to whole numbers. ^1^ Evaluated at end of follow-up.

**Table 3:** Risk of pharmacological or hospital treatment for depression in 1324 patients with colorectal cancer compared with 6620 cancer-free comparison persons.

Time Since Inclusion	Model 1 ^1^ HR (95% CI)	Model 2 ^2^ HR (95% CI)	Model 3 ^3^ HR (95% CI)
0–1 year	13.50 (8.89–20.51)	13.06 (8.59–19.68)	12.01 (7.89–18.28)
1–3 years	9.08 (6.17–13.36)	9.09 (6.13–13.51)	8.40 (5.65–12.49)
3–5 years	6.25 (3.90–10.01)	6.18 (3.86–9.90)	5.77 (3.60–9.25)
More than 5 years	2.73 (1.66–4.47)	2.80 (1.70–4.60)	2.65 (1.61–4.36)

^1^ Adjusted for sex, age at inclusion, time since enrollment in the Diet, Cancer and Health Cohort. ^2^ Adjusted for 1 + education, smoking, alcohol consumption, Body Mass Index and MET-score. ^3^ Adjusted for 1 + 2 + Charlson Comorbidity Index score, cohabitation and stage.

**Table 4:** Lifestyle, clinical and treatment related risk factors for depression for 1324 patients with colorectal cancer.

-	Person Years	No Events	Model 1 ^1^ HR (95% CI)	Model 2 ^2^ HR (95% CI)	Model 3 ^3^ HR (95% CI)
Lifestyle ^4^					
Smoking					
Current	1821	75	1.33 (0.93–1.89)	1.03 (0.91–1.86)	1.29 (0.90–1.86)
Former	1920	61	1.09 (0.75–1.58)	1.07 (0.74–1.56)	1.07 (0.73–1.57)
Never	1807	55	reference	reference	reference
Alcohol consumption per week ^5^					
Abstainers	354	11	0.82 (0.44–1.52)	0.83 (0.45–1.54)	0.89 (0.48–1.66)
Moderate use	4286	147	reference	reference	reference
Excessive use	877	32	1.08 (0.73–1.59)	1.11 (0.75–1.64)	1.18 (0.79–1.76)
Body Mass Index (kg/m^3^)					
<25	2121	78	reference	reference	reference
>25–30	2516	80	0.88 (0.64–1.21)	0.85 (0.61–1.17)	0.89 (0.64–1.23)
>30	913	33	0.98 (0.65–1.47)	0.91 (0.60–1.38)	1.03 (0.67–1.57)
Physical activityMET-score in quartiles ^6^					
1st quartile (least active)	1314	53	1.30 (0.87–1.95)	1.33 (0.88–1.99)	1.23 (0.81–1.86)
2nd quartile	1601	50	0.98 (0.65–1.48)	1.02 (0.68–1.54)	1.04 (0.69–1.59)
3rd quartile	1279	44	1.08 (0.71–1.64)	1.11 (0.72–1.70)	1.16 (0.76–1.79)
4th quartile (most active)	1355	43	reference	reference	reference
Charlson Comorbidity Index Score ^7^					
0	3307	84	reference	reference	reference
1–2	1625	67	1.77 (1.28–2.46)	1.72 (1.24–2.40)	1.74 (1.24–2.43)
3+	617	40	2.73 (1.86–4.02)	2.63 (1.79–3.88)	2.74 (1.84–4.09)
Clinical factors					
Cancer type					
Colon	3326	120	reference	reference	reference
Rectum	2223	71	0.96 (0.71–1.30)	0.94 (0.70–1.28)	0.90 (0.65–1.23)
Stage					
1	1224	32	reference	reference	reference
2	2055	50	0.90 (0.57–1.40)	0.90 (0.58–1.40)	0.88 (0.56–1.38)
3	1591	50	1.14 (0.73–1.78)	1.12 (0.72–1.76)	1.10 (0.70–1.73)
4	449	53	3.08 (1.96–4.84)	3.10 (1.97–4.88)	3.07 (1.95–4.83)
Treatment					
Surgery					
no	337	23	reference	reference	reference
yes	5212	168	0.77 (0.48–1.26)	0.77 (0.47–1.24)	1.28 (0.74–2.24)
Surgical complications					
no	4474	134	0.72 (0.44–1.17)	0.71 (0.44–1.16)	1.20 (0.68–2.11)
yes	738	34	1.12 (0.63–1.97)	1.09 (0.62–1.93)	1.64 (0.87–3.08)
Stoma					
no	4253	125	0.71 (0.44–1.17)	0.71 (0.44–1.17)	1.13 (0.64–2.01)
yes	959	43	1.03 (0.59–1.784	0.99 (0.58–1.74)	1.61 (0.88–2.96)
Radiotherapy					
no	4863	150	reference	reference	reference
yes	686	41	2.29 (1.60–3.27)	2.27 (1.59–3.24)	2.76 (1.82–4.19)
Chemotherapy					
no	3608	111	reference	reference	reference
yes	1941	80	1.42 (1.05–1.92)	1.42 (1.05–1.93)	0.98 (0.67–1.42)

^1^ Adjusted for sex, age at inclusion, time since enrollment in the Diet, Cancer and Health Cohort. ^2^ Adjusted for 1 + education. ^3^ Adjusted for 1 + 2+ stage + cancer type. ^4^ At time of enrollment in the Diet, Cancer and Health Cohort. ^5^ Based on recommendations from the Danish Health Authorities at time of enrollment. Moderate use, 1–14 drinks per week for women and 1–21 drinks per week for men. Excessive use, >14 drinks per week for women and >21 drinks per week for men. ^6^ The metabolic equivalent of task, i.e., a measure of energy cost of physical activities. Calculated as kcal/kg/hour from an average of summer and winter physical activity multiplied by number of hours per week. 1st quartile: <=14.5 (least active), 2nd quartile: 14.5–26, 3rd quartile: >26–43.8, 4th quartile (most active): >43.8. ^7^ Comorbidity is classified according to a modified the Charlson Comorbidity Index (CCI), excluding cancer.

## Data Availability

The supporting data are not publicly available due to research participant privacy restrictions.

## Supplementary Materials

The following are available online at https://www.mdpi.com/article/10.3390/cancers13081979/s1, Table S1: Lifestyle, clinical and treatment related risk factors for depression for 1324 patients with colorectal cancer with stage I–III, Table S2: Lifestyle related risk factors for depression for 1324 patients with colorectal cancer using information from the Danish Colorectal Cancer Group database at time of diagnosis.
