# Supplementary material for: Risk of Pharmacological or Hospital Treatment for Depression in Patients with Colorectal Cancer–Associations with Pre-Cancer Lifestyle, Comorbidity and Clinical Factors
Source: Cancers (Basel). 2021 Apr 20;13(8):1979. doi: 10.3390/cancers13081979 (PMC8073925; doi:10.3390/cancers13081979)
Supplement: Supplementary file 1 [file cancers-13-01979-s001.zip › cancers-1176590-supplementary.pdf]

Risk of Pharmacological or Hospital Treatment for Depression in Patients with Colorectal Cancer–Associations with Pre-Cancer Lifestyle, Comorbidity and Clinical Factors

Trille Kristina Kjaer, Ida Rask Moustsen-Helms, Vanna Albieri, Signe Benzon Larsen, Thea Helene Degett, Anne Tjønneland, Christoffer Johansen, Susanne K. Kjaer, Ismail Gogenur and Susanne Oksbjerg Dalton

Table S1. Lifestyle, clinical and treatment related risk factors for depression for 1048 patients with colorectal cancer with stage I–III

-	Person years	No events	Adjusted[1] HR (95 % CI)
Lifestyle[2]			
Smoking			
Current	1586	53	1.51 (0.97–2.34)
Former	1689	44	1.20 (0.76–1.89)
Never	1594	35	reference
Alcohol consumption per week[3]			
Abstainers	325	7	0.70 (0.32–1.52)
Moderate use	3756	99	reference
Excessive use	763	25	1.29 (0.82–2.04)
Body Mass Index (kg/m^3)			
<25	1817	55	reference
>25 and <30	2226	50	0.72 (0.48–1.07)
>30	827	27	0.98 (0.61–1.57)
Physical activity			
MET score in quartiles[4]			
1st quartile (least active)	1165	33	1.21 (0.76–1.95)
2nd quartile	1149	25	0.91 (0.57–1.48)
3rd quartile	1408	36	0.80 (0.47–1.36)
4th quartile (most active)	1147	38	reference
Charlson Comorbidity Index[5]			
0–1	2907	54	reference
2	1429	45	1.67 (1.11–2.52)
≥3	534	33	3.17 (2.02–4.98)
Clinical factors			
Cancer type			
Colon	2946	83	reference
Rectum	1924	49	0.96 (0.67–1.39
Stage			
1	1224	32	reference
2	2055	50	0.91 (0.58–1.42)
3	1591	50	1.15 (0.73–1.79)
Treatment			
Surgery[7]			
no	-	-	reference
yes	-	-	1.68 (0.49–5.78)
Surgical complications[7]			
no	-	-	1.56 (0.45–5.39)
yes			2.30 (0.63–8.31)
Stoma[7]			
no	-	-	1.50 (0.43–5.21)
yes	-	-	2.31 (0.64–8.35)
Radiotherapy			
no	4279	105	reference

yes	591	27	2.48 (1.48–4.17)
Chemotherapy			
no	3323	89	reference
yes	1547	43	1.03 (0.66–1.61)

[1] Adjusted for sex, age at inclusion, time since enrollment in the Diet, Cancer and Health Cohort, education, disease stage and cancer type.[2] At time of enrollment in the Diet, Cancer and Health Cohort.[3] Based on recommendations from the Danish Health Authorities at time of enrollement. Moderate use, 1-14 drinks per week for women and 1-21 drinks per week for men. Excessive use, >14 drinks per week for women and > 21 drinks per week for men. [4] The metabolic equivalent of task, i.e., a measure of energy cost of physical activities. Calculated as kcal/kg/hour from an average of summer and winter physical activity multiplied by number of hours per week. 1st quartile: <=14.5 (least active), 2nd quartile: 14.5 – 26, 3rd quartile: >26 – 43.8, 4th quartile (most active): >43.8.[5] Comorbidity is classified according to a modified the Charlson Comorbidity Index (CCI) excluding cancer.[7] Information not shown due to low frequencies.

Table S2. Lifestyle related risk factors for depression for 1324 patients with colorectal cancer using information from the Danish Colorectal Cancer Group database at time of diagnosis.

-	Person Years	No Events	Adjusted[1] HR (95 % CI)
Lifestyle			
Smoking			
Current	739	26	1.05 (0.64–1.70)
Former	1865	59	1.00 (0.68–1.47)
Never	1586	56	reference
Alcohol consumption per week (units)			
Abstainers	458	21	1.02 (0.62–1.68)
1–14	2570	90	reference
15–21	542	16	0.98 (0.56–1.69)
>21	506	13	0.79 (0.42–1.49)
Body Mass Index (kg/m^3)			
<25	1981	75	reference
>25 and <30	1561	42	0.82 (0.56–1.22)
>30	684	26	1.08 (0.68–1.70)

[1] Adjusted for sex, age at inclusion, education, cancer stage and cancer type.

Publisher's Note: MDPI stays neutral with regard to jurisdictional claims in published maps and institutional affiliations.

© 2021 by the authors. Licensee MDPI, Basel, Switzerland. This article is an open access article distributed under the terms and conditions of the Creative Commons Attribution (CC BY) license (http://creativecommons.org/licenses/by/4.0/).
